# LncRBase: An Enriched Resource for lncRNA Information

**DOI:** 10.1371/journal.pone.0108010

**Published:** 2014-09-18

**Authors:** Sohini Chakraborty, Aritra Deb, Ranjan Kumar Maji, Sudipto Saha, Zhumur Ghosh

**Affiliations:** Bioinformatics Centre, Bose Institute, Kolkata, India; National Center for Biotechnology Information, United States of America

## Abstract

Long noncoding RNAs (lncRNAs) are noncoding transcripts longer than 200 nucleotides, which show evidence of pervasive transcription and participate in a plethora of cellular regulatory processes. Although several noncoding transcripts have been functionally annotated as lncRNAs within the genome, not all have been proven to fulfill the criteria for a functional regulator and further analyses have to be done in order to include them in a functional cohort. LncRNAs are being classified and reclassified in an ongoing annotation process, and the challenge is fraught with ambiguity, as newer evidences of their biogenesis and functional implication come into light. In our effort to understand the complexity of this still enigmatic biomolecule, we have developed a new database entitled “LncRBase” where we have classified and characterized lncRNAs in human and mouse. It is an extensive resource of human and mouse lncRNA transcripts belonging to fourteen distinct subtypes, with a total of 83,201 entries for mouse and 133,361 entries for human: among these, we have newly annotated 8,507 mouse and 14,813 human non coding RNA transcripts (from UCSC and H-InvDB 8.0) as lncRNAs. We have especially considered protein coding gene loci which act as hosts for non coding transcripts. LncRBase includes different lncRNA transcript variants of protein coding genes within LncRBase. LncRBase provides information about the genomic context of different lncRNA subtypes, their interaction with small non coding RNAs (ncRNAs) viz. piwi interacting RNAs (piRNAs) and microRNAs (miRNAs) and their mode of regulation, via association with diverse other genomic elements. Adequate knowledge about genomic origin and molecular features of lncRNAs is essential to understand their functional and behavioral complexities. Overall, LncRBase provides a thorough study on various aspects of lncRNA origin and function and a user-friendly interface to search for lncRNA information. LncRBase is available at http://bicresources.jcbose.ac.in/zhumur/lncrbase.

## Introduction

Once set aside as genomic ‘junk’, the non coding repertoire of the transcriptome has steadily emerged to be functionally significant guiding factors in the regulation of various biological processes impacting cellular development, differentiation, and metabolism. Among these, long noncoding RNAs (lncRNAs) have recently become the hotspot of attention due to their remarkable similarity with protein coding associates: they undergo splicing and are most often endowed with 'poly(A) tail [1], [2], a feature hitherto associated with protein-coding transcripts. LncRNAs are transcripts longer than a somewhat arbitrary cut-off of 200 nucleotides (nts) [3], [4], albeit less conserved than protein coding RNAs and have high tissue specificity [5]–[7], thus initially raising the doubt of being ‘transcriptional artifacts’. However, tiling array studies of the human genome point out to the fact that a large fraction of the *transcription machinery* is employed for synthesis and maintenance of lncRNAs [8], [9]. LncRNAs contribute to a plethora of cellular regulatory processes, ranging from X chromosome inactivation, genomic imprinting and chromatin modification, to telomere elongation, transcriptional activation, and nuclear trafficking [10], [11]. Parallel studies on lncRNA function and expression in different cellular systems have led to the accumulation of massive amounts of experimental results, ready to be collated into comprehensive, reliable catalogs of lncRNA information.

With the advent of new technologies achieving unprecedented depths in RNA sequencing, several thousands of lncRNAs have been identified across the mammalian genome with diverse genomic context and mechanistic details [10], [12]–[14]. Employing a combination of *in silico* and wet bench techniques, several independent and collaborative efforts have put forward an impressive catalogue of lncRNAs, with primary emphasis on human and mouse transcripts. lncRNAdb [15] has assembled a list of lncRNAs with referenced information about their biological functions and expression in different systems. The GENCODE consortium [16] has been an extensive resource for human lncRNAs till now, and has recently announced their first catalogue of mouse lncRNAs. The NONCODE database (v3.0 and v4.0) [17], [18] has also grouped together a significant number of human and mouse lncRNAs and provided associated information on lncRNA cellular localization, function and expression. Other recent databases hosting discrete information about different aspects of lncRNAs in human include LNCIpedia [19], lncRNome [20] and LncRNADisease [21]. Despite such extensive work on lncRNAs, there remain certain domains which have not been well defined regarding lncRNA biology and function: one such area is to analyze the influence of different regulatory elements on the function of lncRNAs and *vice versa*. Little is known regarding the regulatory interactions between lncRNA and other small ncRNA classes. Recent reports have suggested that lncRNAs could potentially interact with other classes of ncRNAs and modulate their functions [22]. Further, lncRNAs can act as precursors for small RNAs and can regulate gene expression via small RNA dependent mechanism [22]. Comprehensive information on lncRNA association with Repeat Elements of distinct Repeat Families, with Imprinted gene loci, and distribution of CpG Islands (CGI) in lncRNA promoter regions is still lacking. These are crucial aspects to consider while unraveling the functional complexity of lncRNAs, and would help us delve deep into yet unexplored depths of the cellular regulome. Such incompleteness in existing information on lncRNAs motivated us to analyze these aspects of lncRNA and develop LncRBase. Here we have extensively categorized human and mouse lncRNAs and also featured non coding transcript variants of protein coding genes, like retained introns, processed transcripts, and ambiguous ORF containing non coding transcript variants, obtained from Ensembl (Gene 75).

LncRBase is a comprehensive and user friendly database, with a total of 216,562 transcript entries. The database hosts information on basic lncRNA transcript features, with additional details on their genomic location, overlapping small ncRNAs, association with Imprinted genes, and association of Repeat Elements with each transcript. LncRNA promoters have also been classified based on their association with CGIs. Furthermore, a subset of microarray probes has been remapped to lncRNAs and has been associated with gene expression signatures of specific disease types. The database also hosts lncRNA expression data obtained from RNA-sequencing studies in different tissues from human and mouse. This would provide further insight into lncRNA function with respect to their expression in different tissue systems. Overall, LncRBase will serve as a useful resource for both computational and experimental biologists to browse, search and retrieve information on human and mouse lncRNAs.

## Results And Discussion

### Distribution of different lncRNA subtypes based on their genomic location

**Figure 1:**
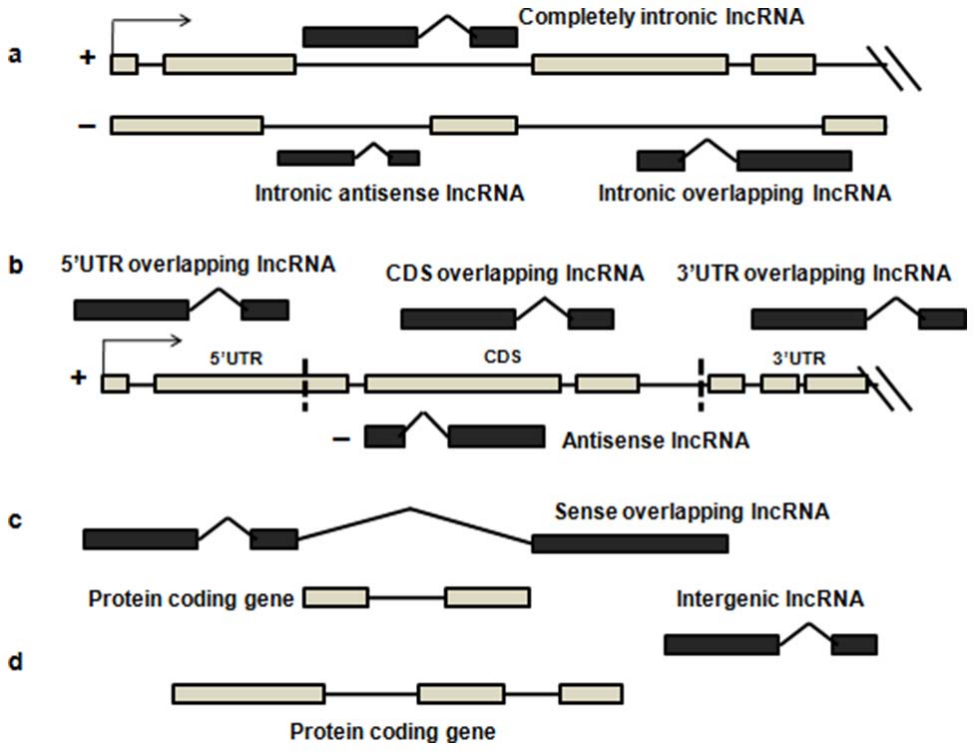
Diagram of the genomic context of different lncRNA subtypes. (a) CI: Completely Intronic lncRNA, IA: Intronic Antisense lncRNA and IO: Intron Overlapping lncRNA; (b) 3UO: 3/UTR Overlapping lncRNA, 5UO: 5/UTR Overlapping lncRNA, CD: CDS overlapping lncRNA and AN: Antisense lncRNA; (c) SO: Sense Overlapping lncRNA; (d) LI: Intergenic lncRNA;

LncRBase hosts fourteen distinct subtypes of lncRNAs including our newly classified types and other transcript biotypes. These are as follows:

(1) 3UO, 3/UTR overlapping lncRNAs overlapping any 3/UTR exon in the sense strand. (2) 5UO, 5/UTR overlapping lncRNAs overlapping any 5/UTR exon in the sense strand. (3) CDS, CDS overlapping lncRNAs overlapping any CDS exon. (4) LI, Intergenic (linc) lncRNAs transcribed from in between two gene loci. (5) AN, Antisense lncRNAs intersecting any exon of a protein-coding locus on the opposite strand. (6) CI, Completely Intronic transcripts residing within introns of a coding gene, but do not intersect any exons. (7) IA, Intronic Antisense lncRNAs completely overlapping with an intron in the opposite strand.(8) IO, Intron Overlapping lncRNA splice variants of a gene, contain intronic sequence.(9) PS, Pseudogene transcripts having homology to protein coding transcripts but containing disrupted coding sequence and an active homologous gene can be found at another locus.(10) SO, Sense Overlapping lncRNAs containing a coding gene in its intron on the same strand. (11) AO, Ambiguous ORF transcripts believed to be protein coding, but with more than one possible open reading frame.(12) PT, Processed Transcripts not containing an ORF (obtained from Ensembl dataset).(13) MI, miscRNA from the Ensembl transcript dataset. (14) NC, Non coding transcripts not falling in any of the above mentioned categories. Diagrammatic illustration of the subtypes are given in **Figure 1**.

LncRNAs are known to be greatly varied in length, starting from the popular consensus of 200 bps upto ∼9 Kb. Since transcript length influences their secondary structure formation and functional variation [23], [24], hence to show the length distribution of human and mouse lncRNAs we have plotted the corresponding length distribution graph [**Figure S1**]. We observe that most lncRNAs fall within the 500–1,000 bp length window, in both the organisms.

**Figure 2:**
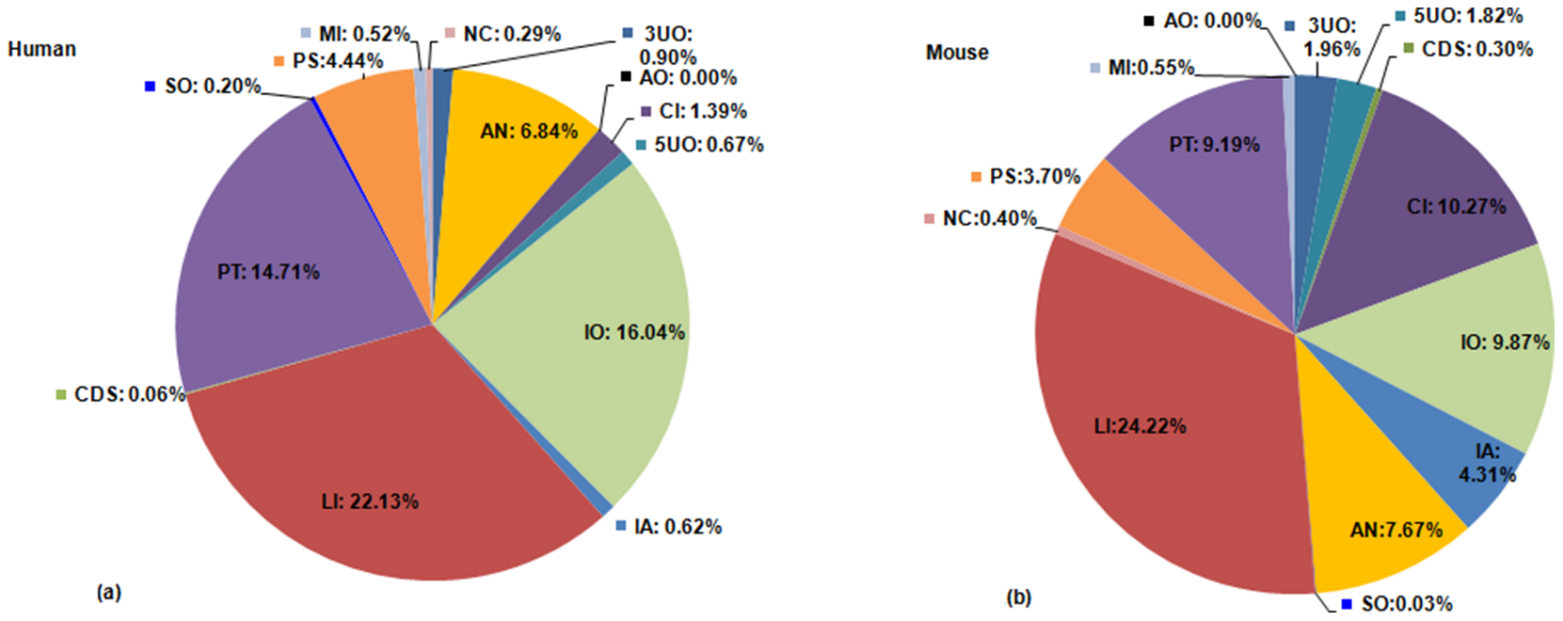
Distribution of non coding lncRNA subtypes. Distribution of noncoding lncRNA subtypes in (a) human and (b) mouse. Abbreviations: (1) 3UO, 3/UTR overlapping lncRNAs (2) 5UO, 5/UTR overlapping lncRNAs (3) CDS, CDS overlapping lncRNAs (4) LI, Intergenic (linc) lncRNAs (5) AN, Antisense lncRNAs (6) CI, Completely Intronic (7) IA, Intronic Antisense lncRNAs (8) IO, Intron Overlapping lncRNA (9) PS, Pseudogene lncRNAs (10) SO, Sense Overlapping lncRNAs (11) AO, Ambiguous ORF transcripts (12) PT, Processed Transcripts (13) MI, miscRNAs (14) NC, Non coding transcripts.

Distribution of non coding lncRNA (i.e. with no coding potential) subtypes follows the pattern presented in **Figure 2**(**a–b**). LIs are the most abundant type of lncRNAs in human followed by PT lncRNAs [**Figure 2**(**a**)]. In mouse, majority of lncRNAs fall in the LI type, followed by CI lncRNAs [**Figure 2**(**b**)]. Inclusion of FANTOM3 transcripts in the dataset might be the reason for the abundance of intron-associated transcripts in mouse [25]. Abundance of intergenic (LI) lncRNAs reflects the extent of annotation of different subtypes of lncRNAs, intergenic lncRNAs being extensively categorized in previous works.

### Ambiguous lncRNAs overlap with multiple genomic elements

Some lncRNAs map to more than one genomic locus and have been grouped accordingly under two or more subtypes. These lncRNAs can have more than one type of association with different coding gene elements, like introns and exons. An example would include uc021ssq.1(a UCSC transcript), which is both AN and IO. The ID assigned to such lncRNA transcript having dual identity is hsaLB_AN_88674.1 and hsaLB_IO_88674.1 with a change only in the ‘subtype’ part of the identifier [AN and IO]. Due to such genomic context, these lncRNAs are open to multiple interpretations and have been grouped as ‘Ambiguous lncRNAs’. 3,578 human lncRNAs (2.7% of total transcripts) and 3,731 mouse lncRNAs (4.7% of total transcripts) show such ambiguous behaviour. These lncRNAs can be separately searched in the database and have also been provided in **Data S1**.

We have also considered non coding transcript variants of protein coding genes and included them in our list of lncRNAs. 47,598 and 23,124 newly predicted human and mouse lncRNAs respectively have been obtained from the retained intron (included in our Intron Overlapping subtype), ambiguous ORF and processed transcript variants of protein coding genes in Ensembl (Gene75). A detailed list of such transcripts and corresponding genes can be found in **Data S2**.

### Coding potential of lncRNAs

It has been stated that certain genes have bifaceted transcript outputs that participate in distinct spectrums of gene regulatory interactions. A well known example would be SRA1, which shows bifunctionality, both as an RNA regulator and a functional protein encoder [26], mediated by alternative splice variants. The protein-coding longer SRA1 isoforms include the same core sequence as needed for the regulatory lncRNA function which is thus concluded to be bi-functional. This necessitates careful examination of the coding capability of a putative non-coding transcript before it could satisfy such paradigm of bifunctionality. We have calculated the coding potential of each transcript using Coding-Potential Assessment Tool (CPAT) [27]. In order to imply a selective inclusion criteria for specifying an lncRNA, we have separated transcripts showing a positive CPAT coding probabilty score and grouped them as ‘putatively coding’ transcripts which would warrant further stringent investigations. Human transcripts with Coding Probability (CP) score <0.364 were declared noncoding and those with CP> = 0.364 were declared putatively coding. CP threshold used for mouse was 0.44 (CP<0.44 was non coding and CP> = 0.44 was putatively coding). CP threshold values considered for calculating non coding and putatively coding transcripts were as per CPAT documentation. Since many lncRNAs reported so far have not yet undergone experimental validation regarding their protein coding capabilities, this will serve as a reference score for users to select and analyze specific lncRNAs based on their coding potential.

**Figure 3:**
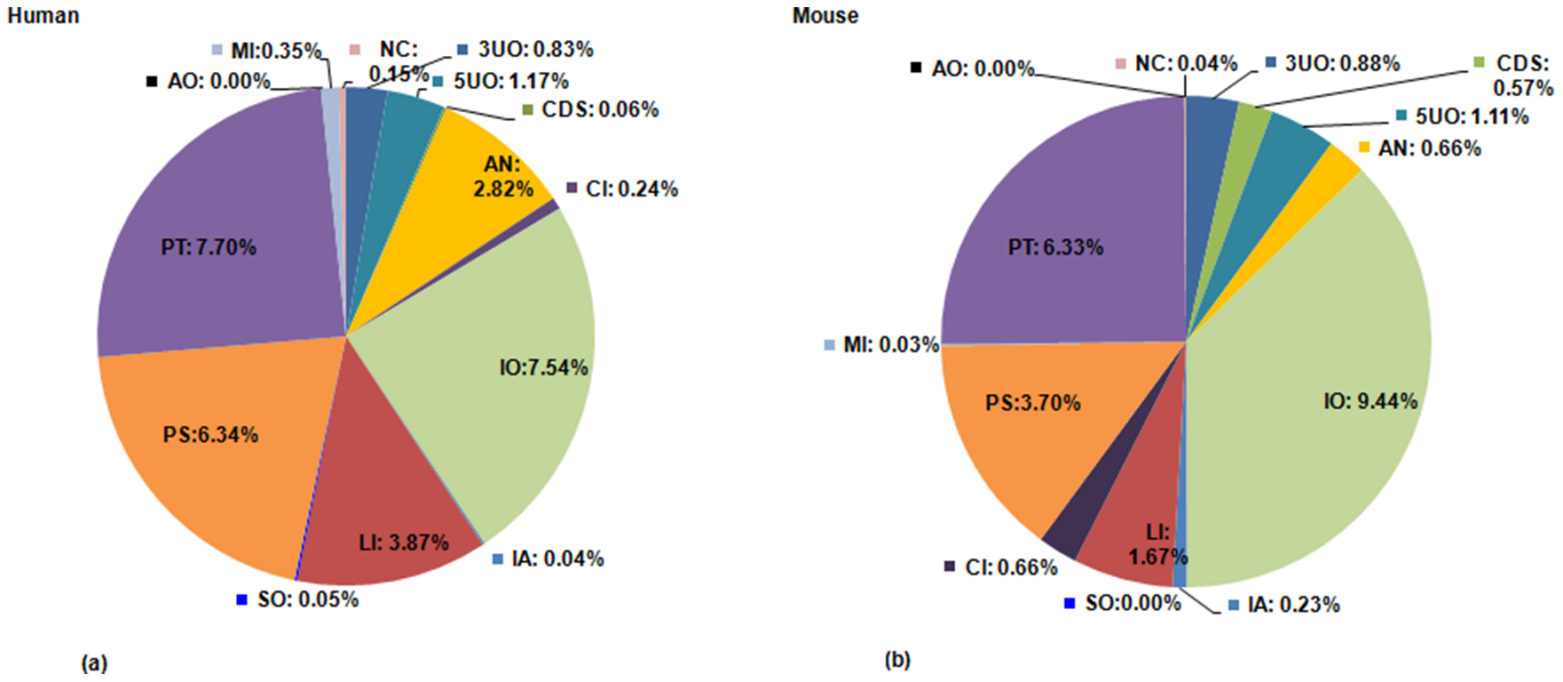
Distribution of putatively coding lncRNA subtypes. Distribution of putatively coding lncRNA subtypes in (a) human and (b) mouse. Abbreviations: (1) 3UO, 3/UTR overlapping lncRNAs (2) 5UO, 5/UTR overlapping lncRNAs (3) CDS, CDS overlapping lncRNAs (4) LI, Intergenic (linc) lncRNAs (5) AN, Antisense lncRNAs (6) CI, Completely Intronic (7) IA, Intronic Antisense lncRNAs (8) IO, Intron Overlapping lncRNA (9) PS, Pseudogene lncRNAs (10) SO, Sense Overlapping lncRNAs (11) AO, Ambiguous ORF transcripts (12) PT, Processed Transcripts (13) MI, miscRNAs (14) NC, Non coding transcripts.

**Figure 3**(**a–b**) demonstrates subtype wise distribution of putatively coding lncRNAs in human and mouse. It is logical to assume that some of these transcripts might exhibit a bifunctional mode of operation, with dual role as a regulator and a messenger, since some lncRNAs have been reported to have such a functional dichotomy [28], [29]. However, since recent investigations have redefined the coding capacity of previously annotated lncRNA transcripts with stringent, high-confidence annotation protocols [30], these putatively coding lncRNA transcripts would serve as a preliminary dataset for sorting out potential transcripts with proposed dual mode of function, which have been wrongly tagged with the ‘ncRNA’ moniker.

### Association of lncRNAs with small ncRNAs

LncRNAs interact in a well-regulated and orchestrated manner with various biomolecules to participate in a multi-layered integrated regulatory circuitry. It has been in reports recently that lncRNAs harbour small ncRNAs [8]. miRNAs are a class of small ncRNAs which are ∼22 nt short endogenous RNAs that comprise of the most highly explored class of gene regulatory molecules in multicellular organisms [31]. They not only function as microregulators of protein-coding genes but also interact with and regulate the functions of different ncRNAs [32]. Large scale transcriptome analyses have pointed out several sites of miRNA-lncRNA interaction across the genome [33]. LncRNA-miRNA interaction acts as an additional strata in the regulatory interactome, where lncRNAs act as miRNA quenchers and promote gene expression and subsequent functional manifestation [34]. piRNAs are small ncRNAs of 25–33 nts in length, that are derived from transposable elements within the genome. They are involved in cellular epigenetic programming via pairing with piRNA-complementary binding sites in the genome which act as guidance cues for the recruitment of epigenetic factors in target sites [35]. LncRNAs associated with germline specific piRNA clusters during male germline development may function to regulate gene expression via piRNA-mediated epigenetic mechanisms [36].

Based on such reports we sought to find out a positional preference for small RNA abundance viz. piRNA and miRNA abundance within certain lncRNA loci.

miRNA associated lncRNAs: 2,624 human and 941 mouse lncRNAs mapped with miRNA primary transcripts in the same strand within human and mouse genome respectively. Subsequently deepBase [37] annotated small RNA clusters (deepBase contains small RNA sequencing data from multiple experiments) were mapped to these primary miRNAs. We considered the lncRNA associated primary miRNA transcripts which constitutes the small RNA clusters. A Significance Score (for an lncRNA transcript *j*) was assigned to assess the primary miRNA transcript abundance within that lncRNA locus (details on Significance Score is provided in Materials and Methods section).

piRNA associated lncRNAs: Human and mouse piRNAs were mapped to human and mouse genome respectively and piRNA clusters were computed following the definition of Lau *et al* [38] (discussed in Materials and Methods section). These piRNA clusters were mapped to lncRNA transcripts which resulted in 1,302 and 2,547 piRNA cluster-associated lncRNA transcripts in human and mouse respectively. A Significance Score (for an lncRNA transcript *j*) was calculated, based on the number of piRNAs (constituting a particular piRNA cluster) to assess the piRNA abundance within that particular lncRNA locus (details on Significance Score is provided in Materials and Methods).

Overall, these miRNA and piRNA associated lncRNAs can harbour such small ncRNAs and the Significance Score representing the abundance of mature piRNAs or primary miRNAs within a particular lncRNA reveals the plausibility of biogenesis of these small RNAs from the lncRNA transcript.

### Associated Repeat Elements

**Figure 4:**
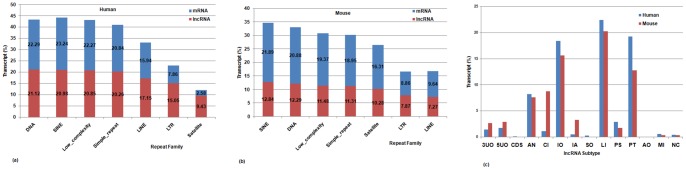
Distribution of Repeat Elements across lncRNAs. Distribution of different Repeat Families across lncRNAs in (a) human and (b) mouse compared to protein coding transcripts. (c) Repeat associated lncRNA subtypes in human and mouse. Abbreviations: (1) 3UO, 3/UTR overlapping lncRNAs (2) 5UO, 5/UTR overlapping lncRNAs (3) CDS, CDS overlapping lncRNAs (4) LI, Intergenic (linc) lncRNAs (5) AN, Antisense lncRNAs (6) CI, Completely Intronic (7) IA, Intronic Antisense lncRNAs (8) IO, Intron Overlapping lncRNA (9) PS, Pseudogene lncRNAs (10) SO, Sense Overlapping lncRNAs (11) AO, Ambiguous ORF transcripts (12) PT, Processed Transcripts (13) MI, miscRNAs (14) NC, Non coding transcripts.

LncRNAs containing Alu repeats can participate in post-transcriptional regulation of protein coding RNAs through imperfect base pairing with 3/UTR Alu elements and targeting mRNA transcripts for Staufen-mediated decay (SMD). These lncRNA-3/UTR base-pairing interactions create double-stranded STAU1-binding sites in mRNA 3/UTRs, inducing Staufen 1 (STAU1) binding, resulting in destabilization and degradation of the target mRNA [39], [40]. Repeat containing lncRNAs are also known to take part in translational regulation of an mRNA. SINEB2 Repeat Element containing lncRNAs complement with 5/UTR of mRNAs in a head-to-head fashion and upregulate translation. Well known examples include mouse Uchl1AS lncRNA and antisense KCS1 lncRNA in yeast [39]. A mutated L1 element in an lncRNA is associated with infantile encephalopathy [41]. HERVH or Human endogenous retrovirus subfamily H is a class of transposable elements essential for both development and maintenance of pluripotency in somatic cells [42]. All these evidences point out the functional versatility of Repeat-associated lncRNAs and the importance of repetitive sequences in lncRNA transcripts. Several lncRNAs contain functional repeat sequence domains [43] and lincRNAs have been shown to contain a significant proportion of highly repetitive transposable elements (TE) [44]. Hence, we mapped Repeat Elements (belonging to different Repeat Families) to lncRNA loci and analyzed the distribution of Repeat Elements in individual lncRNA transcripts. **Figure 4**(**a–b**) shows the significance of the association of Repeat Elements with lncRNAs compared to that with protein coding transcripts. SINE and DNA Repeat Families are more abundant in lncRNAs in both human and mouse compared to other Repeat Families. Among the different lncRNA subtypes, lincRNAs (LI) are the most abundant class of Repeat-associated lncRNAs both in human and mouse [**Figure 4**(**c**)]. This is in line with previous observation that intergenic lncRNAs tend to be associated with transposable elements [44]. Processed Transcript (PT), Intron Overlapping (IO) and Antisense (AN) lncRNAs also show a significant association with Repeat Elements in both human and mouse. Overall, lncRNA-Repeat associations would provide further insight into yet another facet of genome regulation via noncoding mediators containing repeat domains [45].

### Associated Imprinted genes

Imprinted gene clusters contain one or several lncRNAs functioning as *cis*-acting silencers of neighbouring protein coding genes [46]. LncRNAs were mapped to Imprinted genes in order to find out overlapping transcripts. A total of 918 and 415 lncRNA-imprinted gene associations were found in human and mouse respectively.

Imprinted ncRNAs show different imprinting features compared to imprinted protein-coding genes, and have a greater participation in the mechanism of genomic imprinting. These imprinted ncRNAs coexist with large imprinted regions in the genome and act as key players in the evolution and regulation of genomic imprinting [47]. This provides further insight into the function of lncRNAs as potential regulators of imprinting and the expression of other genes associated with imprinted loci.

### lncRNA promoter analysis

**Figure 5:**
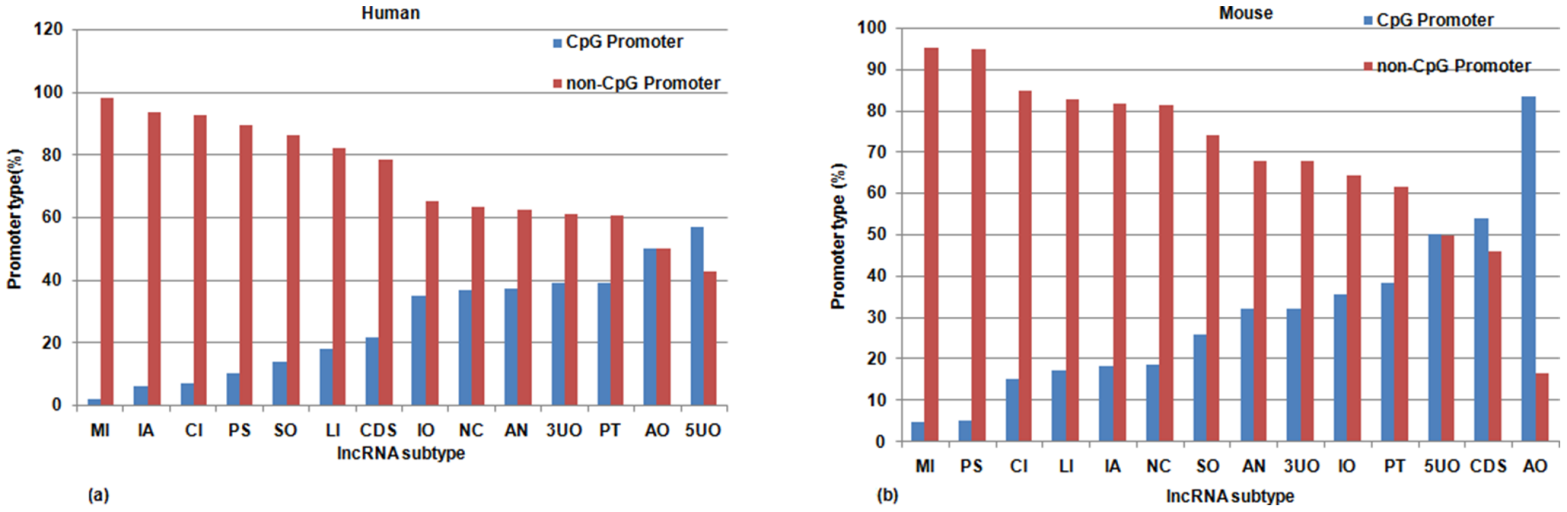
Distribution of CGI and non-CGI promoters among the various lncRNA subtypes. CGI and non-CGI promoters of each subtype of lncRNA (a) in human (b) in mouse. Abbreviations: (1) 3UO, 3/UTR overlapping lncRNAs (2) 5UO, 5/UTR overlapping lncRNAs (3) CDS, CDS overlapping lncRNAs (4) LI, Intergenic (linc) lncRNAs (5) AN, Antisense lncRNAs (6) CI, Completely Intronic (7) IA, Intronic Antisense lncRNAs (8) IO, Intron Overlapping lncRNA (9) PS, Pseudogene lncRNAs (10) SO, Sense Overlapping lncRNAs (11) AO, Ambiguous ORF transcripts (12) PT, Processed Transcripts (13) MI, miscRNAs (14) NC, Non coding transcripts.

CpG Islands (CGIs) are generically equipped to influence local chromatin structure and regulate gene activity. CGI promoters have their own distinctive chromatin configuration and show specific patterns of transcription initiation [48]; modifications like cytosine methylation in the CpG moieties themselves result in stable shutdown of the associated promoter [48]. 45% of all human gene promoters, particularly the tissue specific gene promoters, do not lie within CGIs [49]. These non-CGI promoters are subjected to DNA methylation, regulating the establishment and maintenance of tissue-specific expression patterns [50]. LncRNA promoter regions (−1 to +1 kb from TSS) of each lncRNA transcript in mouse and human were mapped with CGIs to classify them as CGI or non-CGI promoters. We found 35,674 and 19,957 CGI lncRNA-promoters in human and mouse respectively. We have recorded the CGI name, %GC content, CpG density and type of overlap with the lncRNA promoter region. Most of these CGI promoters showed a high CpG content, indicative of distinct functional status of the lncRNA transcripts. Non-CGI promoters are more abundant in lncRNAs: 93,144 and 58,646 non-CGI promoters were found in human and mouse respectively. **Figure 5**(**a–b**) gives a distribution of CGI and non-CGI promoters of each biotype of lncRNA in human and mouse genomes.

Abundance of non-CGI promoters is consistent across most of the lncRNA subtypes in both human and mouse. Given the evidence regarding prevalence of non-CGI promoters in establishment of tissue-specific gene expression patterns, this feature corroborates their association with lncRNA transcripts, which are also prone to maintain a distinct tissue-specific expression profile. An exception is noted in case of exon overlapping (5UO, CDS, AO) lncRNA subtypes where abundance of CGI promoters point towards the possibility of divergent transcription from protein coding gene promoters [51] giving rise to non coding lncRNA isoforms.

### Microarray probe classification and disease association

Recent studies point out that certain lncRNA sequences uniquely map with conventional, pre-annotated microarray probesets. Hence, available microarray data can be mined to obtain lncRNA expression profiles [52]–[54]. 4,631 Affymetrix GeneChip Human Genome U133 Plus 2.0 Array probes and 2,707 Affymetrix GeneChip Mouse Genome 430 2.0 Array probes in human and mouse respectively were remapped to lncRNA transcripts. From Gene Expression Barcode [55], [56], we obtained consensus gene expression signatures of different tumor tissues in human and mouse. A binary version of expression values or a ‘barcode’ is assigned to provide expression calls for all genes on the Affymetrix GeneChip Human Genome U133 Plus 2.0 Array and Affymetrix GeneChip Mouse Genome 430 2.0 Array. Based on this barcode, probes are assigned an expression call of 1 or 0 to denote expressed and silenced calls respectively based on their expression in a particular tissue type. Probes remapped to lncRNAs were finally matched to these probes associated with different tumor tissue specific consensus gene expression datasets. These disease-associated lncRNAs would serve as a starting dataset for subsequent experiments which are essential to conclude about the actual state of expresssion of these lncRNAs in respective disease systems.

### lncRNA expression in tissues

We examined the expression patterns of human and mouse lncRNAs in different tissues. To this end, we downloaded RNA-Seq dataset comprising of 12 cell lines in human (GSE30567) and 6 tissues in mouse (GSE30352) from Gene Expression Omnibus (http://www.ncbi.nlm.nih.gov/geo/) and estimated lncRNA expression in these tissues. Spliced alignment was carried out using TopHat2 [57] and lncRNA transcript expression was thus estimated (in FPKM) using Cufflinks [58]. Analysed lncRNA expression data for different tissues is available in our database.

### Search and output options

**Figure 6:**
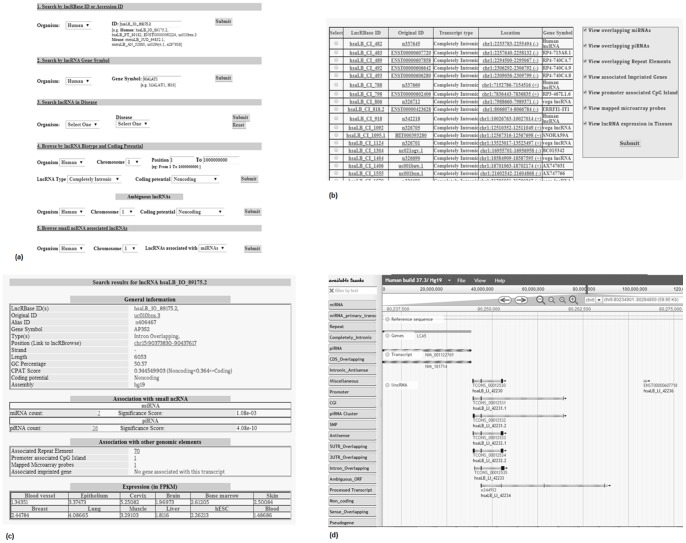
Different web interfaces allow easy view of lncRNA information. (a) Search page provides multiple options for searching lncRNA information. (b) General Output page displays basic information about an lncRNA transcript and provides multiple options for probing into further details. (c) Detailed Output page displays complete information about an lncRNA transcript. (d) LncRBrowse allows user to browse through different genomic annotation tracks.

LncRBase primarily processes the user query through simple search options, which in turn retrieve information from the relational database tables, format the result and display it on the web interface [**Figure 6**(**a–c**)]. Six different search options are provided by which a user can look for lncRNA transcript information which are as follows:

1. Search by lncRNA Accession ID: User can input specific lncRNA Accession ID a) from LncRBase like hsaLB_IO_89175.2 for human and mmuLB_AN_52880 for mouse or from b) any of the source databases (Ensembl Gene75, UCSC ID, NONCODE v3.0, H-InvDB 8.0 or Human Body Map lincRNAs) to view detailed information about that transcript.
2. Search by lncRNA Gene Symbol: User can input known lncRNA Gene Symbol to search for the number of transcript entries for that gene recorded in LncRBase and associated literature references for that gene; clicking on any LncRBase ID will direct the query to a webpage containing detailed information about that particular transcript.
3. Search lncRNA in Disease: Selecting any particular disease will show the probes associated with a consensus expression profile for that particular disease type and the lncRNAs mapped to each of these probes. Further experiments in future are to be done to check the actual expression of these lncRNAs in the corresponding disease states.
4. Browse by lncRNA subtype and coding potential: Any specific lncRNA subtype can be selected to view basic information about the corresponding lncRNAs. Given specific genomic locus as input, the ouput will display all lncRNAs mapped in that position. One can add an extra search option by which they can check lncRNA transcripts as per their coding potential.
5. A separate section within the fourth search option allows to check for Ambiguous lncRNA transcripts which belong to multiple subtypes. User can give a chromosome wise search based on coding potential.
6. Search for ncRNA associated lncRNAs: User can check for lncRNAs associated with small ncRNAs viz. primary miRNAs and piRNAs by a chromosome wise search in respective genomes.
7. Search lncRNA expression: ‘Search lncRNA expression’ page allows the user to view expression of lncRNA transcripts (belonging to different subtypes), obtained from RNA-Seq data within different tissues in human and mouse.

### Visualizing LncRBase

For viewing the information related to lncRNAs, a browser (lncRBrowse) has been integrated. This browser provides an integrated view of Refseq Genes, Refseq Transcripts, lncRNA subtypes, lncRNA Promoter, CpG Islands, Repeat Masker 3.27 Repeats, miRBase (v20) primary miRNA transcripts and mature miRNAs, piRNAs and SNPs from dbSNP. Browsing through these tracks would allow the user to check for their association with respect to each other [**Figure 6**(**d**)]. Details of each annotation track has been provided as a pdf in Browse and Help page of LncRBase and also as **Data S3**.

## Materials and Methods

### Data procurement

All transcript sequence information corresponds to human hg19 and mouse mm10 genome assemblies respectively. The cDNA sequences for both mouse and human ncRNAs were obtained from different sources which are listed in **Table 1** **.** Since input file formats were different such as fasta, bed and gff3, we have developed custom scripts for extraction of sequences and annotations, which are stored in our database. Genomic elements, including 5/UTR exons, 3/UTR exons, CDS exons, introns (RefSeq annotations), CGIs and fasta sequences of lncRNA promoter regions were downloaded from the University of Santa Cruz (UCSC) Table Browser data retrieval options. miRNA related information was downloaded from mirBase20 [59]. piRNA sequences were downloaded from National Centre for Biotechnology Information (NCBI) [60] in fasta format. Imprinted genes and their annotations were downloaded from Geneimprint [http://www.geneimprint.com]. For re-annotating the microarray probes, we downloaded Affymetrix GeneChip Human Genome U133 Plus 2.0 Array and Affymetrix GeneChip Mouse Genome 430 2.0 Array probe sequences from manufacturer's website [http://www.affymetrix.com]. Consensus gene expression signatures for different tumor tissues were downloaded from Gene Expression Barcode database for different tumor tissues [55], [56]. RNA-Seq datasets were downloaded from NCBI Gene Expression Omnibus (GEO) [61]. For human, we downloaded the ENCODE Cold Spring Harbor Lab Long RNA-seq data (GSE30567) and for mouse we used PolyA+ RNA-Seq data from GSE30352. For curating available literature on lncRNAs, we downloaded all PubMed evidences from NCBI using the keyword ‘RNA’ and then screened out relevant records using gene name search.

**Table 1:** Source of transcripts for LncRBase.

Database	Version	Organism	Number of transcripts
**Ensembl ** [16]	Gene 75	Human	93753
		Mouse	36069
**UCSC Genome Browser database ** [68]		Human	15321
		Mouse	15141 (noncoding transcripts)
**NONCODE ** [17]	v3	Human	33801
		Mouse	36991
**Human bodymap lincRNAs ** [14]		Human	14353
**H-InvDB ** [69]	8.0	Human	20395 (noncoding transcripts)
**Total set of nonredundant transcripts**			128818 (human)
			78603 (mouse)

### Data processing and refinement

#### A. Redundancy check and assigning Alias ID

**Figure 7:**
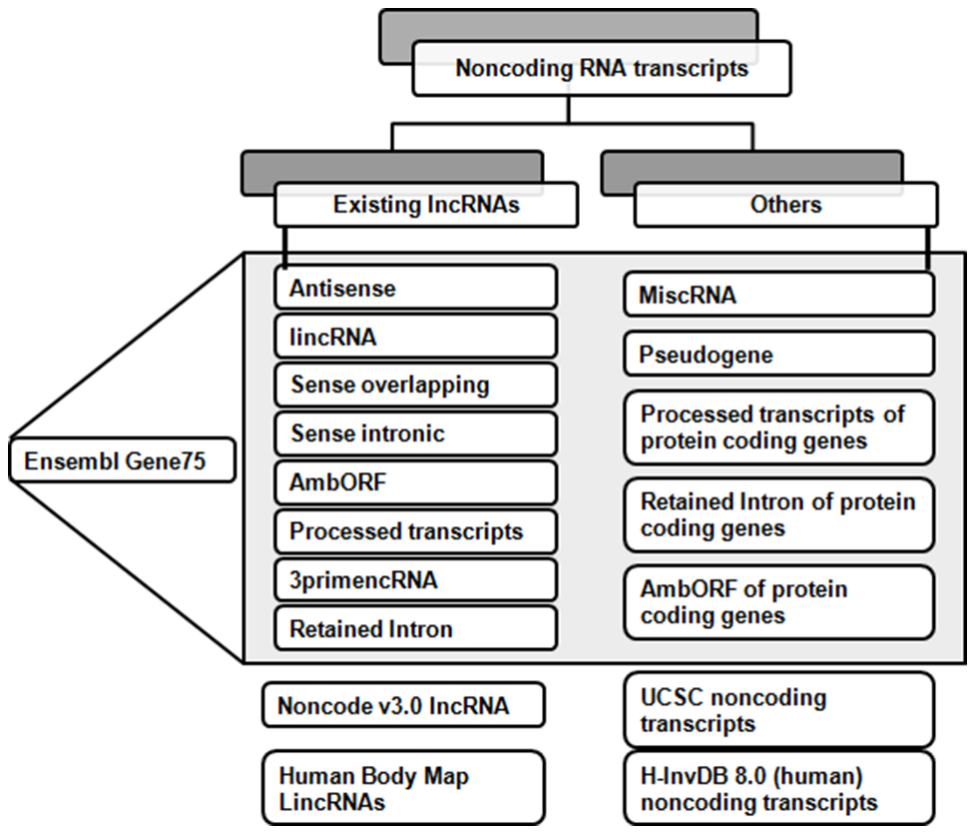
Classification scheme for LncRBase transcripts. The transcripts selected were either pre-annotated as lncRNAs or simply mentioned as non coding transcripts. In the former case, additional details were provided about the transcripts, while in the latter case, transcripts > = 200 nts were selected and characterized as lncRNAs.

Initial sets of ncRNA transcripts taken from different sources [**Table 1**] were classified into two different types [**Figure 7**] based on their existing annotation. The already annotated lncRNAs were grouped into ‘Existing lncRNAs’. The rest were grouped as ‘Others’. This second group of ncRNA transcripts was filtered based on the length of the transcript sequences, with a cut off of ≥ 200 nts to comply with the basic criteria of an lncRNA transcript.

The resultant dataset needed further refinement for redundancy removal since the data sources mentioned above contain a varying degree of overlapping data. To decide whether two primary entries might represent the same lncRNA, we considered their chromosomal location and sequence similarity as reference. LncRNAs were aligned to each other using BLAST-Like Alignment Tool (BLAT) [62] with default parameters. We considered alignments having block count = 1 and mismatch = 0. We calculated percent-overlap of the both the reference and the query transcript sequence. Transcripts having more than 99% sequence similarity and overlap in genomic coordinates were considered to be ‘Identical’. The rest of the transcripts did not have any redundant entry. The ‘Identical’ transcripts were finally kept as one unique entry in LncRBase and assigned a unique LncRBase ID. The redundant transcripts were mentioned as ‘Alias IDs’.

#### B. Classification of lncRNAs and assignment of unique transcript IDs

All the lncRNA transcripts were classified and annotated according to their location, with reference to protein-coding gene elements in their respective genome. This was done by comparing their chromosomal coordinates to those of a comprehensive list of pre-annotated genomic elements, including 5/UTR exons, 3/UTR exons, CDS exons, and introns.

Based on the chromosomal location, we have assigned a unique identifier to each transcript. The lncRNA transcripts have been designated a unique identifier as [abbreviation of species name] LB_ [*subtype*] _ [*number*]. Abbreviation of human and mouse are ‘hsa’ and ‘mmu’ respectively. LB is the abbreviation of LncRBase, ‘*subtype*’ is the distinct subtype of an lncRNA transcript. The ‘*number*’ is assigned to the transcript based on its positional/sequential occurrence within the genome.

E.g. :(a) hsaLB_CI_482 implies a human lncRNA transcript whose subtype is Completely Intronic (CI) and its sequence of occurrence within the human genome is 482.

(b) mmuLB_CI_7 implies a mouse lncRNA transcript whose subtype is Completely Intronic (CI) and its sequence of occurrence within the mouse genome is 7.

For each of the cDNA transcript variants which have the same chromosomal locus, the number is extended by a numerical index Eg: IDs hsaLB_CI_8429.1 and hsaLB_CI_8429.2 imply that there exist two cDNA transcript variants from the same genomic locus.

#### C. Assessment of coding capacity

Standalone Coding-Potential Assessment Tool (CPAT) [27] was used to check the coding probability of the lncRNA transcripts. CPAT has high accuracy (0.967) and efficiency (10,000 times faster than CPC [63] and PhyloCSF [64]). For human, a coding probability threshold of 0.364 was used as cut-off. Transcripts with Coding Probability (CP) score <0.364 were declared non coding and those with CP> = 0.364 were declared putatively coding. CP threshold used for mouse was 0.44 (CP<0.44 was non coding and CP> = 0.44 was putatively coding). CP threshold values considered for calculating non coding and putatively coding transcripts were as per CPAT documentation. Briefly, nonparametric two-graph ROC curves are used to determine an optimal CPAT score threshold that maximizes the discriminatory power and a score threshold of 0.364 gave the highest sensitivity and specificity (0.966 for both) for human data [27]. The CPAT score threshold of 0.44 was calculated similarly for mouse.

#### D. Mapping piRNAs and miRNAs to lncRNAs

piRNA associated lncRNAs: Human piRNA sequences reported by Girard *et al* [65] and mouse piRNA sequences reported by Girard *et al* [65] and Lau *et al* [38] were obtained from NCBI [60] and mapped to human and mouse genome respectively. As piRNAs are known to originate in the system as piRNA clusters, possible piRNA clusters were computed following the definition of Lau et al [38] (minimum piRNA density of 20 per Kbase, window span of 20 Kbases and window increment of 1 Kbase). LncRNA transcripts were mapped to these piRNA clusters. Based on the number of piRNAs (constituting a particular piRNA cluster) occurring within an lncRNA transcript locus, a Significance Score (for an lncRNA transcript *j*) was calculated to assess the piRNA abundance within that particular lncRNA locus as given by the following formula:
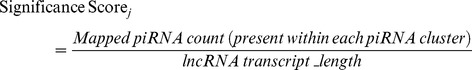



miRNA associated lncRNAs: Primary miRNA sequences obtained from miRBase v20 [59] were mapped to the lncRNA transcripts. Subsequently deepBase [37] annotated small RNA clusters (this database contains small RNA sequencing data from multiple experiments) were mapped to primary miRNA associated lncRNA transcripts. Based on the number of small RNA reads (constituting each RNA cluster) that mapped to these lncRNA transcripts, the Significance Score (for an lncRNA transcript *j*) was calculated to assess the primary miRNA abundance within that lncRNA locus as is given by the following formula:
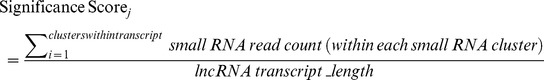



Significance Score, represents the abundance of piRNAs or primary miRNAs within a particular lncRNA transcript.

#### E. Remapping of microarray probes to lncRNAs

Affymetrix GeneChip Human Genome U133 Plus 2.0 Array and GeneChip Mouse Genome 430 2.0 Array probe sequenes were aligned to lncRNA sequences using BLAT with default parameters. Alignment results were filtered by a criteria of block count = 1, mismatch = 0, match size = query size, strand =  negative. Filtered probes were selected to be mapped to lncRNAs.

#### F. RNA-Seq expression study

For RNA-Seq data, all sequenced reads from each tissue type were aligned to the human and mouse reference genome using the spliced read aligner TopHat2 [57]. Transcript assembly and abundance estimation of each tissue type was performed using Cufflinks [58].

### Implementation

All computational programs for the collection, sorting and redundancy removal of the data and the genome mapping of putative lncRNA transcripts to exons, introns, miRNA primary transcripts, piRNA, Imprinted genes, CGIs were executed using custom Perl scripts and UNIX shell scripting languages.

**Figure 8:**
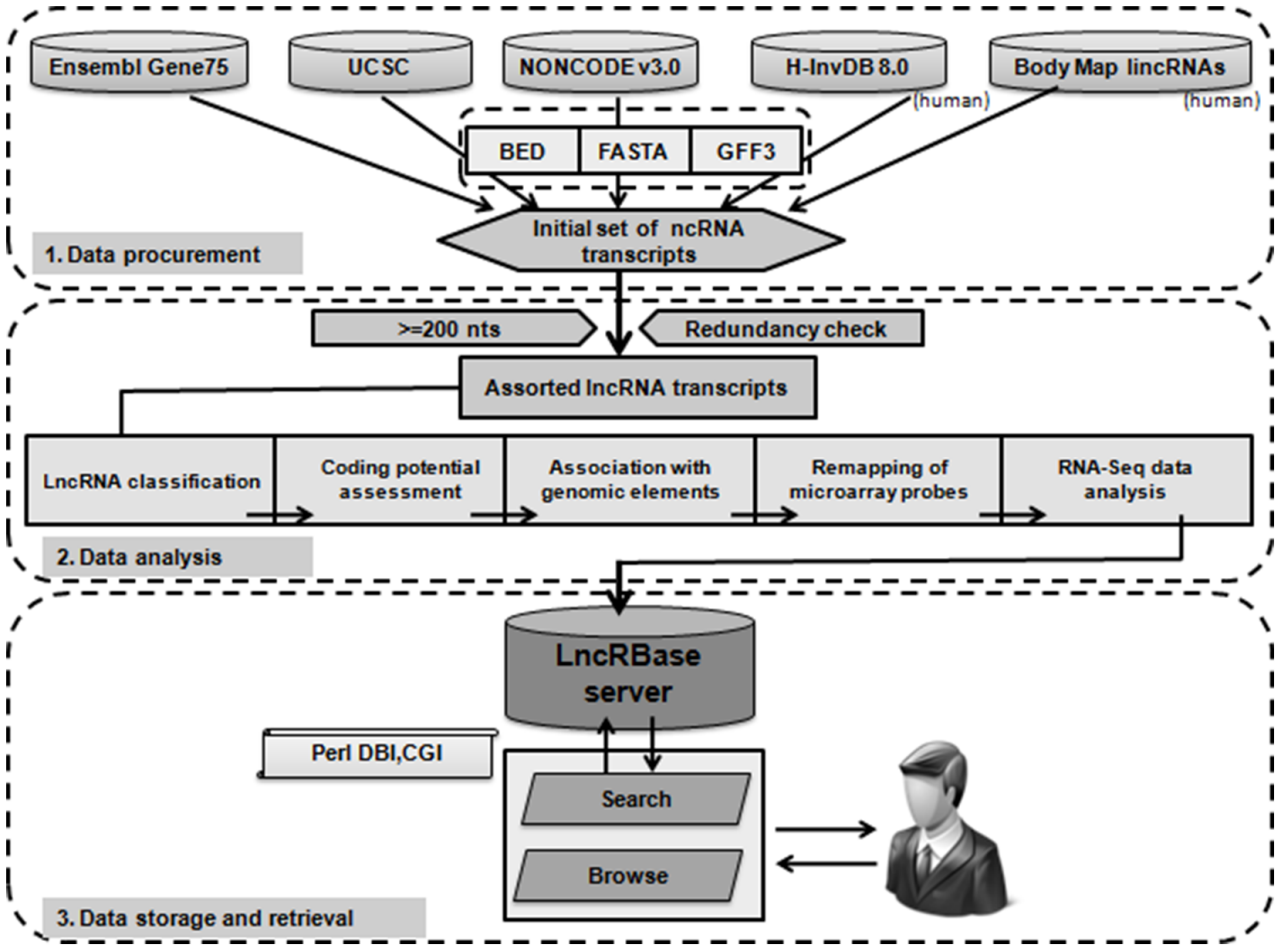
Workflow diagram of LncRBase. LncRBase is compiled by subsequent importing, naming, analysis and visualization of lncRNA transcripts. Every lncRNA transcript is subsequently characterised using multiple parameters, and the results are stored in the database. A web-interface built using Perl enables lncRNA visualization and database querying.

LncRBase has been developed as a relational database using MySQL. This web server runs in a Linux environment. The search engine is powered by apache http deamon. The interface layer has been designed using HTML/CSS and the database is connected to the web interface using perl CGI module. Server side Perl scripts are implied to connect and query LncRBase using perl DBI module, and to generate dynamic HTML pages to produce output [**Figure 8**]. LncRBrowse has been implemented using JBrowse 1.9.8 [66], [67], and the browse interface of LncRBase is connected to lncRBrowse using php script.

## Conclusion

The discovery of several thousands of lncRNAs and current upsurge in lncRNA annotation and characterization has added another layer of complexity towards understanding mammalian genomes and transcriptomes. Given the ever increasing number of transcripts identified as lncRNAs, it has not yet been possible to precisely define the functional repertoire of these versatile transcripts. A combination of *in silico* and laboratory-based approaches is needed to analyze lncRNA biogenesis and their various functional intricacies. Our contribution to the rapidly expanding field of ‘lncRNomics’ has been directed towards constructing a well collated lncRNA catalog incorporating our findings and those of other published works in the form of a comprehensive database. We have classified lncRNAs based on their genomic position relative to known protein-coding genes. We have analyzed the association of lncRNAs with Repeat Elements, CGIs, Imprinted genes, small ncRNAs like primary miRNAs and piRNAs. Microarray probe sets have been remapped to lncRNAs and associated with different disease systems. LncRNA expression information has also been provided which will help towards understanding tissue-specific behaviour of these multifunctional transcripts.

LncRBase will serve as an enriched resource for lncRNAs with respect to data and information content. Six important features are key points of LncRBase: (i) elucidating non coding transcript variants of protein coding genes, (ii) usage of a unique identifier for each lncRNA transcript, (iii) analysis of lncRNA promoter regions, (iv) association of lncRNA transcripts with primary miRNAs and piRNAs (v) association of lncRNA transcripts with Imprinted genes and (vi) association of lncRNA transcripts with Repeat Elements. These, along with other detailed information available are expected to make LncRBase a useful resource for lncRNA research in human and mouse systems.

LncRBase integrates information of varied content starting from basic sequence information, extending to categorization based on genomic context, coding potential score, re-annotated microarray probes, associated disease information and lncRNA expression in different tissues in human and mouse. LncRBase is designed to enable integration with other resources, including the UCSC Genome Browser, Ensembl, NONCODE v3.0 and other databases, thus providing an integrated repository for lncRNAs.

With the advances in next generation sequencing technology, more lncRNA genes are expected to be discovered. LncRBase will incorporate these newly annotated lncRNA sequences to update existing information. We plan to incorporate structure based classification information on the lncRNA transcripts. LncRBase has the potential to become a community resource for lncRNA transcript information and annotation.

### Availability

LncRBase is freely available at http://bicresources.jcbose.ac.in/zhumur/lncrbase/. The LncRBase data files can be freely downloaded and used in accordance with the GNU Public License.

## Supporting Information

Figure S1 **Length distribution of human and mouse lncRNAs.**
(TIF)

Data S1 **List of ambiguous lncRNAs in human and mouse.**
(XLSX)

Data S2 **List of non coding transcript variants from protein coding genes.**
(XLSX)

Data S3 **Annotation tracks for LncRBrowse.**
(PDF)
