# Supplementary figures and images for: LncRBase: An Enriched Resource for lncRNA Information
Source: PLoS One. 2014 Sep 18;9(9):e108010. doi: 10.1371/journal.pone.0108010 (PMC4169474; doi:10.1371/journal.pone.0108010)

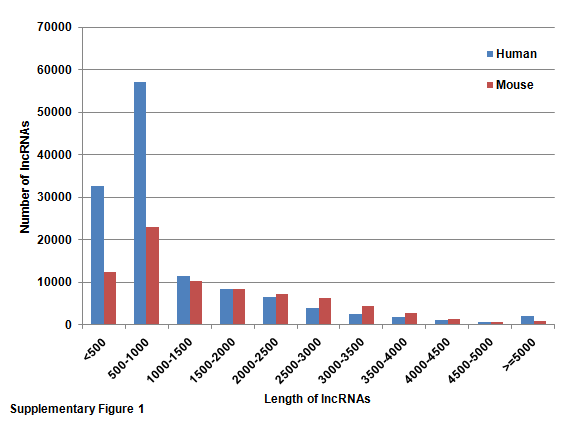

Supplement: Figure S1 — Length distribution of human and mouse lncRNAs. (TIF) [file pone.0108010.s001.tif]
