# Supplementary material for: LncRBase: An Enriched Resource for lncRNA Information
Source: PLoS One. 2014 Sep 18;9(9):e108010. doi: 10.1371/journal.pone.0108010 (PMC4169474; doi:10.1371/journal.pone.0108010)
Supplement: Data S3 — Annotation tracks for LncRBrowse. (PDF) [file pone.0108010.s004.pdf]

Annotated tracks for lncRBrowse

1. **Genes** Refseq genes have been downloaded via UCSC Genome Browser and uploaded as a gene track.

2. **Transcript** Refseq transcripts downloaded via UCSC Genome Browser

3. **Repeat** Repeat Masker 3.27 Repeats downloaded via UCSC Genome Browser

4. **CGI** CpG Islands downloaded from UCSC Genome Browser database. Track provides name and chromosomal position of the CpG Island.

5. **Promoter** Putative promoter region of individual lncRNAs. Regions between -1 to +1 kb from TSS have been considered as promoter region for each lncRNA transcript. Track shows the name of the transcript with which the promoter is related and chromosomal position and length of the putative promoter region.

**6. miRNA primary transcripts**<br>**7. miRNA**	.gff3 files of miRNA primary transcript and mature miRNAs from miRBase 20.Tracks contain information about miRNA name, ID, chromosomal position.

**8. CDS_Overlapping**<br>**9. 3UTR_Overlapping**<br>**10. 5UTR_Overlapping**<br>**11. lincRNA**<br>**12. Ambiguous_ORF**<br>**13. Antisense**<br>**14. Intronic_Antisense**<br>**15. Miscellaneous**<br>**16. Pseudogene**<br>**17. Sense_overlapping**<br>**18. Intron_Overlapping**<br>**19. Completely Intronic**<br>**20. Processed_Transcript**<br>**21. Non_coding**	lncRNA tracks. Fourteen separate tracks have been created for fourteen subtypes of lncRNA as per LncRBase categorisation protocol. Individual tracks contain information about LncRBase ID, original ID, type, chromosomal position and exons of each lncRNA.

22. **piRNA** Total piRNA information like piRNA name, piRNA ID (NCBI ID), Chromosomal position (annotated by our in-house pipeline) and length have been provided as a track option to view via lncRBrowse.

23. **SNP** Total SNPs downloaded from NCBI dbSNP. Type of allele change, class of SNP and chromosomal position of the SNP is shown. User can view SNPs with specific subtype of lncRNA to see what SNPs fall within the lncRNA locus.
